# Pediatric respiratory co-infection and immunologic response: peds recon study protocol

**DOI:** 10.1038/s41390-025-04509-9

**Published:** 2025-10-24

**Authors:** Milissa U. Jones, Emily L. Parsons, Priscilla A. K. Kobi, Alison M. Helfrich, David King, David L. Saunders, Allison M. W. Malloy

**Affiliations:** 1 Department of Pediatrics, Uniformed Services University of the Health Sciences, Bethesda, MD USA; 2 The Henry M. Jackson Foundation for the Advancement of Military Medicine Inc., Bethesda, MD USA; 3 Department of Internal Medicine, Uniformed Services University of the Health Sciences, Bethesda, MD USA

## Abstract

**Background:**

Acute Viral Respiratory Infections (AVRIs) from SARS-CoV-2, influenza, and RSV are major causes of pediatric illness, yet little is known about how non-medically attended infections influence immune development. Understanding early-life AVRIs patterns and their impact on the developing mucosal immune system is essential to inform interventions that improve long-term child health.

**Methods:**

This ongoing prospective observational cohort study, initiated in January 2024, investigates respiratory viral infections, co-infections, and their effects on immune responses in children aged 1 month to 17 years. Participants are followed for two years, with data collected at baseline (when healthy) and during acute visits (when symptomatic). At baseline, nasopharyngeal swabs and blood samples are collected for viral screening and immune analysis. During acute visits, swabs are collected for viral detection and immunologic assays. If SARS-CoV-2, influenza, or RSV is detected, a post-acute visit occurs 10–21 days after symptom onset for repeat swabs and blood collection. The primary outcome is to define age-based immune responses and assess how co-infections influence immune regulation.

**Discussion:**

This study captures the full spectrum of pediatric AVRIs, including non-medically attended illnesses, to address gaps in immune development research. Findings may inform strategies to optimize prevention and treatment across childhood.

**Impact:**

- Acute viral respiratory infections are the most common cause of childhood morbidity and mortality; however, they are frequently non-medically attended, leaving critical gaps in our understanding.
- Co-infections with respiratory viruses such as SARS-CoV-2, influenza, and respiratory syncytial virus (RSV) may alter immune responses, but their impact on long-term immunity remains poorly understood.
- Understanding age-dependent patterns of respiratory viral infections and co-infections will provide critical insights into immune development and potential vulnerabilities in pediatric populations.
- This study aims to define age-based differences in immunity in the respiratory track in response to respiratory viral pathogens and examine the impact of co-infection on immune regulation.

## Background

Acute Viral Respiratory Infections (AVRIs) are common and costly in pediatric populations, leading to significant healthcare expenditures and disruptions in daily life, including absenteeism from school, work, and childcare.^1–3^ While medically attended AVRIs are well-documented, non-medically attended cases remain underexplored, despite their likely role in viral transmission and long-term respiratory health.^4^ Co-infections, where multiple respiratory viruses infect an individual simultaneously or sequentially, complicate disease progression and are linked to more severe outcomes, including longer hospital stays, ICU admissions, and higher mortality.^5^ Although co-infections such as SARS-CoV-2 and influenza have been shown to increase morbidity in adults,^6,7^ similar data in pediatric populations are limited.^8^

In addition to medical comorbidities, age is an important risk factor for severe disease from respiratory viruses, particularly RSV, influenza, and SARS-CoV-2.^9,10^. Interestingly, this age-dependent pattern is not the same amongst all respiratory viruses. RSV, for example, tends to more frequently cause severe morbidity and mortality in young children compared to SARS-CoV-2.^11^ However, its interaction with other viral pathogens and impact on acute immune response and the development of protective immunologic memory responses remains largely unexplored.

Viral co-infections can influence host immune responses through various mechanisms, including enhanced viral replication, immune modulation, and disruption of immune memory formation. These interactions may impact both protective immunity and the development of harmful inflammatory responses.^12,13^ Understanding how co-infections shape immune responses is critical for informing vaccine development and optimizing strategies to support both short- and long-term respiratory health. Respiratory syncytial virus (RSV), a major cause of respiratory illness in young children, is notable for its high reinfection rates despite minimal antigenic drift at key neutralizing antibody sites.^14^ This paradox underscores the importance of understanding mucosal immunity and raises the question of whether co-infections alter immunologic memory—particularly in light of the COVID-19 pandemic, which disrupted typical viral transmission patterns and seasonality.

The immune system undergoes critical development during early childhood^15^, and early exposures to AVRIs likely influence this process.^16^ Both innate and adaptive immune responses develop in tandem to control infections and establish long-term immunity, though these responses can also contribute to unintended inflammatory effects, such as asthma or wheezing.^17^ The innate immune response, particularly in neonates and young children, plays a crucial role in initial viral control^18^ and immune system maturation, but limited understanding of how early viral infections shape these responses hampers our ability to predict long-term outcomes. Recent studies suggest that upper respiratory tract immune responses, including cytokine and cellular changes, provide valuable insights into how local immune responses are initiated and evolve during infection.^19^ The respiratory system continues to develop through early adolescences^20^, and early life infections can impact the architecture^21^ immune regulation^22^, and microbiome^23,24^ of the respiratory tract resulting impaired lung health.^25^ Understanding the tissue-specific immune response in the developing respiratory tract of pediatric populations exposed to multiple viral infections is essential for elucidating how co-infections modulate immune activation and impact acute and long-term respiratory health.

## Hypothesis

We hypothesize that age-related differences in innate and adaptive immune responses influence viral clearance and immune regulation to SARS-CoV-2, influenza, and RSV. Furthermore, co-infection alters immune regulation by inducing either synergistic or antagonistic immune signaling, potentially exacerbating or mitigating disease severity depending on host age.

## Aims and objectives

### Primary objective

To define age-based differences in innate and adaptive immunity against SARS-CoV-2, influenza, and RSV, and to examine the impact of co-infections on immune regulation.

### Secondary objectives

1. To define differences in the innate immune activation profile associated with SARS-CoV-2, influenza, and RSV single infection compared to co-infection in pediatric age cohorts.
2. To define the adaptive T cell and humoral response upon single SARS-CoV-2, influenza, and RSV infection versus co-infection in pediatric age cohorts.
3. To determine the frequency of respiratory viral co-infections in a post-pandemic cohort, and the impact of SARS-CoV-2, influenza, and RSV.

## Methods

### Study design

**Fig. 1:**
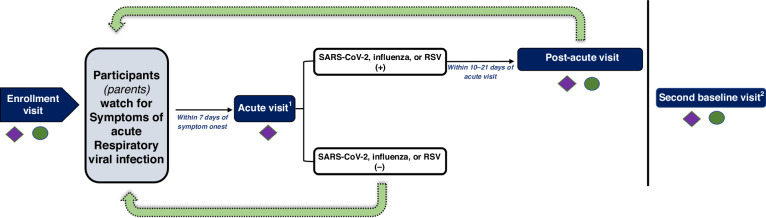
Sequence of study visits and biospecimen procurement in the pediatric respiratory co-infection and immunologic response study (Peds RECON). ^1^The Acute Visit sequence may recur no sooner than 2 months after the previous Acute Visit throughout the duration of the 2-study enrollment. ^2^The Second Baseline Visits occurs at 12 months after the Enrollment Visit only if participants have not been seen for any acute respiratory viral infections in the previous 12 months.  Indicates nasopharyngeal sample collection.  Indicates peripheral blood sample collection.

This is a longitudinal, prospective, observational cohort study evaluating patterns of AVRIs in children and their qualitative effects on the immune system by age and virus. Figure 1 illustrates the sequence of study visits. A total of 200 participants will be followed for two years with in-person visits and bio-specimen collection. Enrollment will be staggered over a four-year period, with study activities continuing for five years to allow for longitudinal follow-up. We plan to enroll 30 participants in Year 1, 60 in Year 2, 60 in Year 3, and 50 in Year 4. This phased approach ensures that the number of active participants at any given time remains manageable, allowing adequate capacity for timely sample collection and data capture during respiratory viral illnesses across all enrolled age groups. Accrual will be on a rolling basis, and dropouts will be replaced.

### Study setting

The study is being conducted at the Uniformed Services University (USU) through the Translational Medicine Unit (TMU), located on the Naval Support Activity (NSA) Bethesda military installation in Bethesda, Maryland. The TMU is equipped with state-of-the-art facilities for conducting clinical research and is dedicated to advancing translational science, particularly in the context of military health. The location facilitates the integration of military-specific considerations and enables recruitment of military-connected children, while also allowing for the enrollment of civilian children, providing a diverse study population. The NSA Bethesda campus is frequented by both children of military and non-military families who work on the installation. The campus also houses two daycare centers and two military medical facilities, including the David E. Cabrera University Family Health Center at USU and the Walter Reed National Military Medical Center, both of which routinely provide pediatric care.

### Sample size calculation

We will recruit up to 220 participants, stratified by age group, to account for an anticipated 10% attrition rate, ensuring a final sample size of 200 participants. The power analysis was based on 3D relationship between power, sample size, and the Cohen’s omega effect size for a Chi-square test with 3 degrees of freedom. This analysis showed that a total sample size of *N* = 200 would be needed to detect Cohen’s omega effect sizes as small as *w* = 0.23 with 80% power. This target may be adjusted if infection rates among study participants differ significantly from expected estimates. This sample size is designed to provide robust statistical power to examine age-based immune responses to SARS-CoV-2, influenza, and RSV, as well as the impact of co-infection on immune regulation. Participants will be divided into age groups as follows:

- **35** children in the 1–11 months cohort
- **65** children in the 1–5-year-old cohort
- **65** children in the 6–11-year-old cohort
- **55** children in the 12–17-year-old cohort

### Eligibility criteria

#### Inclusion criteria

- Age 1 month to 17 years
- Access to NSA Bethesda campus

#### Exclusion criteria

- A history of cancer or an immune system disorder
- A bleeding disorder
- Facial trauma, fracture, or surgery in the past 3 months
- Gestational age less than 35 weeks at birth (if currently under 3 years old)
- Use of monoclonal antibodies in the past 6 months or IVIG in the past 11 months
- A major chromosomal abnormality (e.g., trisomy 21)
- Current pregnancy
- Current use of intranasal steroids, adrenergic agonists, or inhaled corticosteroids
- Immunosuppressive or anti-inflammatory therapy (excluding non-steroidal anti-inflammatory medications) within the past 6 weeks
- Any other medical condition affecting immune function, as determined by a study physician

##### Exclusion criteria rationale

To ensure valid interpretation of immune responses, the study enrolls healthy participants with unaltered immunity. Therefore, individuals with known immunodeficiencies or those receiving immunomodulatory therapies are excluded. A study physician reviews all medical conditions and medications to assess eligibility, and any uncertainty regarding potential immunosuppressive effects is referred to the Principal Investigator for final determination. Additionally, because sample collection involves nasopharyngeal (NP) swabs and peripheral blood draws, individuals with conditions that could interfere with or contraindicate these procedures are excluded.

### Study processes

#### Participant recruitment

Participants are currently recruited through multiple strategies. Approximately twice weekly, study recruiters conduct tabling sessions at the Walter Reed National Military Medical Center Pediatric Immunization Clinic and the Pediatric waiting area, where they engage with parents and families either waiting for appointments or recently completing clinical visits. Recruiters also participate in health fairs and special events held on the NSA Bethesda campus by setting up informational tables and engaging with passersby. Flyers containing study details, contact information, and a link to the study website for self-referral are posted in and around the Walter Reed Pediatrics Clinic, University Family Health Center, outpatient pharmacies, USU, and NSA Bethesda daycare centers. Additional flyers are distributed at other military installations within a 50-mile radius of the research site, as well as community locations—such as public libraries and civilian daycare centers—within a 25-mile radius. Online recruitment methods include paid social media advertisements and listings through the clinical research promotion platform ResearchMatch®.

#### Informed consent

The informed consent process is completed electronically or via paper consent forms. The Informed Consent Document (ICD) and Assent Form were reviewed and approved by the USU Institutional Review Board (IRB) prior to study initiation. The ICD outlines the potential benefits and risks of participation, and states that participation is voluntary, withdrawal is allowed at any time, and refusal involves no penalty. Participants and/or their parent/guardian are provided the opportunity to ask questions to ensure understanding of the protocol prior to signing the ICD. For participants aged 7–17, an Assent Form (with parental/guardian permission) is signed before any study-related procedures. Parental/guardian permission is documented before obtaining assent from minors. For children aged 6 and under, a Waiver of Documentation of Assent is used, confirming the study is explained in age-appropriate terms and that the child either expresses verbal assent or lack of the capacity to do so.

#### Visit reminders and participant retention

Following enrollment, presentation for an Acute Visit is participant-driven. Reminders to contact the study team upon development of respiratory symptoms will include study-specific refrigerator magnets placed in the home and quarterly electronic newsletters summarizing study findings for participants and their parents/guardians. An attrition rate of 10% over the two-year study period has been projected. This estimate is informed by retention data from comparable pediatric longitudinal clinical trial cohorts, including those requiring peripheral blood collection, where attrition has ranged from 2 to 17% (median 8%) under active retention management.^26^ A proportion of our study population will be military-connected, and we recognize that frequent relocations among military families, in addition to general study burden, may increase the risk of attrition. To mitigate these risks, we have incorporated proactive retention strategies. Study staff will conduct routine participant contact (via phone, text, or email) to reinforce engagement, provide anticipatory guidance regarding upcoming visits, and address logistical or clinical concerns in real time. Frequent contact will also allow early identification of families planning to relocate, enabling timely replacement of participants who withdraw. Visit scheduling will remain highly flexible, with early morning and late afternoon availability, and study procedures will be coordinated with routine clinical encounters whenever feasible. These strategies are designed to strengthen rapport with families, reduce barriers to participation, and minimize the impact of anticipated moves, thereby increasing the likelihood of achieving the projected 10% attrition rate and preserving statistical power for both primary and subgroup analyses.

### Sample acquisition

An overview of biospecimen procurement by visit type is depicted in Fig. 1. Participants have a minimum of two study visits: an Enrollment Visit and, for those who do not present with an acute illness within the first year of enrollment, a Second Baseline Visit. The number of Acute Visits depends on how many AVRIs participants develop each year, but Acute Visits do not occur more frequently than every 2 months, resulting in a maximum of six Acute Visits per year in addition to enrollment. Acute Visits specifically associated with SARS-CoV-2, influenza, or RSV infection require an additional Post-Acute visit. At every visit, participants are asked for an update to their health history, immunizations, and the date of their last symptoms of respiratory infection. Participants receive compensation for study-related sample collection, including NP swabs and peripheral blood draws.

#### Enrollment

Participants are enrolled when they are perceived to be in good health and do not meet the study-defined criteria for an acute respiratory viral infection for at least 14 days prior to the enrollment date. After informed consent is obtained, a study physician performs a history and physical exam to confirm eligibility. Two NP swabs are collected for pathogen identification via research-grade multiplex polymerase chain reaction (PCR) and downstream immunologic analysis. Peripheral blood is collected for baseline humoral immunity measures, following minimum risk guidelines.

#### Second baseline visit

Participants are eligible for a Second Baseline Visit if they either report no ARVIs during the 12-month period following enrollment or were unable to present for an acute visit within seven days of respiratory viral symptom onset. This visit includes an updated medical history, vital signs, and the collection of two NP swabs and peripheral blood, following minimum risk guidelines.

#### Acute visits

**Fig. 2:**
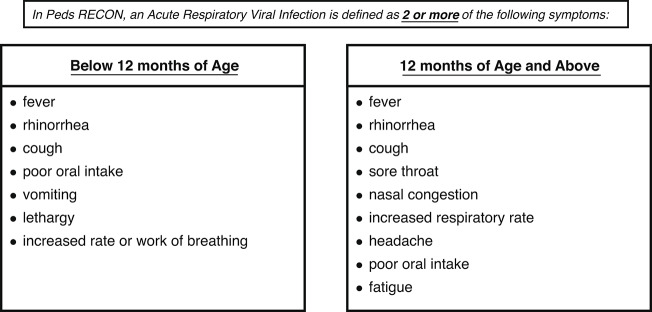
Criteria for acute respiratory viral infection in the pediatric respiratory co-infection and immunologic response study (Peds RECON) by age group.

Participants present for an Acute Visit within seven days of meeting study-defined criteria for an acute respiratory viral infection as defined in Fig. 2. The visit includes an updated medical history, vital signs, and two NP swabs. One swab is pathogen detection via PCR, and the other is used for innate immune analysis and microbiome analysis. Participants receive a paper log to record viral symptoms for 10 days, as well as a follow-up email prompting electronic completion of the symptom questionnaire. If a swab is positive for SARS-CoV-2, influenza, or RSV, then a Post-Acute Visit is scheduled. If negative for those 3 pathogens (even if positive for a different virus), participants continue monitoring for subsequent AVRIs and return for additional Acute Visits, which occur no more than every 2 months. Of note, participants who test positive for pathogens other than SARS-CoV-2, influenza, or RSV, as well as those who test negative for all three, will not have a scheduled Post-Acute Visit.

#### Post-acute visits

Participants with a positive SARS-CoV-2, influenza, or RSV swab from an Acute Visit return for a Post-Acute Visit 10-21 days after symptom onset. This visit includes an updated medical history, vital signs, and the collection of two NP swabs and peripheral blood, following minimum risk guidelines.

### Symptom questionnaire

Following an Acute Visit, participants or their caregivers complete a symptom questionnaire to assess type and frequency of symptoms and assess illness impact (see Supplementary Material S1). Participants or their caregivers rate the severity of 25 symptoms using a Likert scale (0–3, where 3 is severe) daily for 10 days. Additionally, the questionnaire assesses if medication was used for symptomatic relief, when the participant returned to baseline health, if medical attention was sought (and type if applicable), the number of days the participant missed school or daycare, number of days their caregiver missed work, and the number of people in the household who developed symptoms following the participant. Participants receive compensation for completing the symptom questionnaire.

### Sample processing

Two NP swabs are collected per visit. The first NP swab is placed in 3 mL of universal transport media (UTM), the second into 1 mL of UTM. After collection, NP swabs are immediately transported to the lab. Swabs are stored at 4 °C and processed within 1 h of collection. Multiplex PCR testing typically occurs within 2 h of sample collection; however, PCR testing is validated for up to seven days when stored at 4 °C.

Peripheral blood samples are collected in acid citrate dextrose (ACD) tubes. Immediately after drawing the sample, the tube is gently inverted 8–10 times and subsequently stored at room temperature (20 °C) prior to processing.

### Laboratory methods

Specimens collected in this study, including both NP swabs and peripheral blood, undergo comprehensive analyses to evaluate both respiratory viral infections and immune responses. From the NP swab supernatant, 800 ml of universal transport medium is extracted and used for a multiplex PCR assay designed to detect a broad spectrum of upper respiratory tract pathogens, targeting 18 viruses, including SARS-CoV-2, influenza, and RSV, and four bacteria with high sensitivity and specificity overall and for each individual pathogen (see Supplementary Table S2).^27,28^ Any residual supernatant is stored for potential future testing with expanded pathogen panels, allowing for surveillance of emerging respiratory infections as new pathogens are identified.

Cells are isolated from NP swabs through gentle mechanical action and centrifugation. After centrifugation supernatant is removed and stored at −80 °C. Cells from both NP swabs are combined, and an aliquot of cells is taken for analysis of cell surface markers by flow cytometry. The remainder of the cells are cryopreserved in 10% DMSO for single-cell RNA sequencing.

Peripheral blood samples are analyzed for systemic circulating immune responses. Peripheral blood mononuclear cells (PBMCs) and plasma are isolated and cryopreserved for downstream analysis to include measurement of antibody responses and T cell phenotype, and function.

### Clinical data collection and management

Data variables collected in this study are detailed in Table 1. Data obtained at Enrollment, Acute, Post-Acute, and Second Baseline Visits are directly entered or transcribed from paper source documents into electronic case report forms (CRFs) at the study site via Research Electronic Data Capture (REDCap®). Data entered on CRFs, as well as corrections made, are only performed by delegated study staff.

**Table 1:** Variables collected and corresponding timepoints in the pediatric respiratory co-infection and immunologic response study (Ped RECON).

Variable classification	Variable details	Enrollment visit^a^	Acute visit^b^	Post-acute visit^c^	Second baseline visit^d^
Medical history review	○ Past medical diagnoses ○ Surgical history ○ Medications ○ Allergens ○ Immunizations ○ 10 day review of systems	***X***			
Interval medical history review	○ New medical diagnoses ○ New surgical history ○ New medications ○ New allergens ○ New immunizations	**X**	**X**	**X**	**X**
Vital signs	○ Height ○ Weight ○ Heart rate ○ Oxygen saturation ○ Respiratory rate ○ Blood pressure	**X**	**X**	**X**	**X**
Nasopharyngeal testing	○ Respiratory pathogen detection ○ Innate immune analysis ○ Microbiome analysis	**X**	**X**	**X**	**X**
Peripheral blood	○ Quantification of Antibody Responses ○ T cell phenotype ○ T cell function	**X**		**X**	**X**
Symptom questionnaire	○ 10-day review of viral symptom duration and severity ○ Missed days of childcare ○ Caregiver missed days of work ○ Number of healthcare visits ○ Duration and severity of hospitalization (As applicable)		**X**		

*X* Indicates variable collection at this study visit.

^a^Enrollment visit: conducted when participants are healthy and without respiratory viral symptoms for 14 days.

^b^Acute visit: occurs within 7 days of participant experiencing symptoms of a respiratory viral infection

^c^Post-acute visit: conducted within 10–21 days after the Acute Visit when participants are found to be positive with one of the three viruses of interest (SARS-CoV-2, influenza, or Respiratory Syncytial Virus) during the Acute Visit.

^d^Second baseline visit: conducted only when participants have not had an Acute Visit within 12 months post-enrollment.

All data transmitted to and from the application is done using a secure, encrypted transmission

(SSL/HTTPS). For increased security, the application additionally verifies the SSL certificate of the REDCap® server in order to validate the server’s identity. The REDCap® mobile application employs encryption-at-rest on the mobile device’s hard drive; therefore, data and information stored on the device are protected from unauthorized users. REDCap® has built-in safeguards such as username, personal identification number, and remote lockout. Access to study files (e.g., CRFs) is restricted to research team members with task-specific access.

Data collected through this protocol is coded and does not contain personal identifiers. Much of the subsequent research using the coded materials collected under the auspices of this study will be accomplishable through approved exempt or non-human use sub-studies. All research using the materials collected under this protocol is conducted in accordance with an IRB-reviewed protocol, and future analysis will require a new or modified IRB-reviewed protocol. Data and specimens released for research use are labeled by a unique identifier only; data or specimen are shared without any identifiers or links to individuals.

### Ethical approval

This study was approved by the USU Institutional Review Board under protocol number USUHS.2023-121.

### Statistical analysis

The primary objectives are to assess the frequency of viral co-infections in an outpatient pediatric population and to evaluate differences in innate immune responses across infection types, stratified by age group and respiratory pathogen. In the analysis, we will cross-tabulate the frequency of infection/non-infection events into a (2 × 2 × 2 × 4) contingency table having dimensions of SARS-CoV-2, influenza (Yes, No), RSV (Yes, No), and age category (4 groups). The null hypothesis of interest is that the age-category differences in log-odds ratios equal zero for all pairwise differences in age category. We will also perform a Breslow-Day analysis for the homogeneity of for the odds ratios of one infection type across age groups by another infection type. For example, are the odds of influenza infection profile by age the same for RSV-positive and RSV-negative participants?

A secondary analysis will analyze the seasonality of co-infections as a time series. To analyze this data, we will cross-tabulate the frequency of (SARS-CoV-2, influenza, RSV) infection by week. Once this is accomplished, we will perform a seasonal decomposition of the (SARS-CoV-2, influenza, RSV) time-series, which decomposes each time-series into (trend, seasonal, residual) parts. Once each seasonal decomposition is complete, we will then analyze the correlations between the seasonal and trend components of each timeseries. All analyses will be adjusted for age and other relevant covariates, with significance set at *p* < 0.05.

Another analysis will include an omics approach to assess whether differences in means for various inflammatory cytokines or RNA transcriptomic biomarkers vary between infection types (yes/no). To perform this analysis, we will fit a Linear Mixed Effects (LME) model, which uses the collection of inflammatory or RNA transcriptomic biomarkers as the outcome variable. The fixed effect of the model will be a binary indicator of infection for each of the three infections (SARS-CoV-2, influenza, RSV). The random effects of the LME model will be subject-specific intercepts. The null hypotheses are that the (yes-no) difference in mean equals zero. To account for the vast number of hypotheses, the Benjamini-Hochberg procedure will be utilized to maintain the False Discovery Rate (FDR) at 10%.

To analyze whether the mean cytokine levels differ by age group and infection type, we will fit a 4-way ANOVA-type linear mixed effects (LME) model to the data. The response variable in this analysis will be the cytokine level. The fixed effects of this LME model will include age group, SARS-CoV-2 (Yes, No), influenza (Yes, No), and RSV (Yes, No), as well as the two-way interactions of age group with each infection type (Age–SARS-CoV-2, Age–influenza, Age–RSV). The random effects will consist of subject-specific intercepts. The null hypotheses are that the pairwise differences in mean cytokine levels by age group are equal to zero for SARS-CoV-2, influenza, and RSV positive groups. Tukey’s Honest Significant Difference (Tukey’s HSD) method will be used to control the Type I error rate due to multiple hypothesis testing.

## Discussion

This study addresses a critical gap in pediatric infectious disease research by prospectively examining respiratory viral infections—particularly those not medically attended—and their impact on immune development across childhood. Despite widespread documentation of acute viral respiratory infections (AVRIs) that prompt clinical attention, much less is known about the immunologic consequences of infections that do not lead to healthcare encounters. Non-medically attended AVRIs likely account for a substantial proportion of early life viral exposures, yet their role in shaping mucosal and systemic immunity remains poorly characterized. The first year of this study is intentionally structured as a pilot phase to evaluate recruitment feasibility, optimize biospecimen collection procedures, and capture variability in respiratory viral infection patterns. Insights from this initial year will inform modifications to sampling intervals, questionnaire tools, and participant communication strategies, enabling refinement of the full study protocol for future implementation at scale.

Unlike many previous studies that rely on retrospective chart review or clinically captured samples, our prospective, community-based approach allows for observation of AVRIs regardless of medical care-seeking behavior. This offers a more complete view of respiratory viral burden and immunologic responses across the pediatric age spectrum. Although prior investigations have advanced understanding of pediatric immune responses through clinically derived samples,^29,30^ these designs inherently emphasize infections that result in medical encounters and may miss a broader range of mild or asymptomatic cases. In contrast, our protocol recruits participants independent of illness and enables repeated, structured sampling at both healthy and symptomatic timepoints—regardless of healthcare utilization—thereby capturing the full spectrum of infection severity and immune response. This distinction is particularly important in young children, who often experience frequent, mild AVRIs that may nonetheless influence long-term immune trajectories.

Our study objectives focus primarily on characterizing changes in innate and adaptive immunity in response to respiratory viruses. As an exploratory endeavor, we will also leverage nasopharyngeal swabs to examine the broader microbial environment of the respiratory tract, including the bacterial microbiome, that may influence these immune responses. Integration of microbiome analysis offers an avenue to generate hypotheses about how microbial community structure may affect host susceptibility and immune response to viral infections. We hypothesize that viral infection measurably alters the healthy upper respiratory tract microenvironment through effects on both the bacterial microbiome and host immunity. While exploratory, we anticipate that these analyses will provide valuable context for interpreting immunological findings and will inform future research directions. Ultimately, this work has the potential to contribute to the broader scientific understanding of host–microbiome interactions. There are some inherent challenges to our design. Most notably, the frequency of Acute Visits is capped at once every two months to minimize participant burden and ensure feasibility of sample processing. This restriction may result in missed infections that occur in close temporal proximity, potentially underestimating total AVRI frequency. However, this spacing also mitigates the confounding effect of lingering immune responses from immediately prior infections, allowing more interpretable analysis of each AVRI event. In addition, we prioritize detailed immunophenotyping and high-quality sample handling over broader temporal coverage, recognizing the trade-off between depth and breadth in observational cohort studies. While this study is currently limited to a single center and relatively modest cohort size, the pilot nature of the first year will provide critical data on infection rates, immunologic variability, and participant retention, which will inform expansion efforts. To ensure sustainable operations and sufficient coverage of respiratory viral seasons, participant enrollment will occur in a staggered fashion over multiple years, with study activity continuing over five years. This approach helps accommodate the demands of timely Acute Visit sampling and study oversight. However, year-to-year variability in respiratory virus circulation may introduce differences in exposure patterns across cohorts, which could complicate some longitudinal analyses. While this reflects real-world heterogeneity and enhances generalizability, it is an important factor to consider when interpreting findings across the full study population.

The data generated by this protocol will contribute meaningfully to the understanding of age-related immune development in the context of common respiratory viral exposures. By capturing acute and convalescent samples tied to real-world infections, including those not seen in clinical settings, we can identify immune signatures associated with viral clearance, persistence, and potential immune priming or dysregulation. These findings may ultimately inform vaccine design, risk stratification strategies, and interventions that promote healthy immune development. Moreover, this study establishes a flexible, scalable platform for pediatric infectious disease research that can be adapted to emerging respiratory pathogens in future years.

## Supplementary information

Supplementary Material S1

Supplementary Table S2

## Data Availability

Data collection began in January 2024 and is ongoing. Upon completion of the study, data generated and analyzed for this study may be made available upon reasonable request, subject to applicable DoD policies and IRB approval. De-identified data may be shared with qualified researchers upon request and in accordance with relevant data-sharing agreements.
