# Supplementary material for: Pediatric respiratory co-infection and immunologic response: peds recon study protocol
Source: Pediatr Res. 2025 Oct 24;99(6):2206–12. doi: 10.1038/s41390-025-04509-9 (PMC13433244; doi:10.1038/s41390-025-04509-9)
Supplement: Supplementary file 1 [file 41390_2025_4509_MOESM1_ESM.pdf]

# Peds RECON
# Symptom Questionnaire

Please answer the following questions about the participant's current illness. Please follow these instructions:

1. This questionnaire is 4 pages. Complete the Symptom Diary daily.
2. If you do not know if you had the symptom, or are a caregiver filling out the form for your child and do not know if your child has a symptom, you can leave it blank. Please answer each of the 9 questions.
3. An email will be sent to you on Day 11 of illness with a secure link to complete your Symptom Questionnaire in our REDCap Database.

**Questions**:

1. What was the first date the study participant experienced symptoms? (MM/DD/YY)

2. **Symptom Diary:** For the first 10 days of illness, mark on a scale of 0-3 the symptoms experienced:

| 0 | 1 | 2 | 3 |
|---|---|---|---|
| No symptoms | Mild symptoms | Moderate symptoms | Severe symptoms |

Day 1 is the first day the participant began to experience symptoms.
If you do not know the answer, you may leave it blank.

| Symptom | Day 1 | Day 2 | Day 3 | Day 4 | Day 5 | Day 6 | Day 7 | Day 8 | Day 9 | Day 10 |
|---|---|---|---|---|---|---|---|---|---|---|
| Runny or dripping nose | | | | | | | | | | |
| Congestion | | | | | | | | | | |
| Sinus Pressure | | | | | | | | | | |

| Symptom | Day 1 | Day 2 | Day 3 | Day 4 | Day 5 | Day 6 | Day 7 | Day 8 | Day 9 | Day 10 |
|---|---|---|---|---|---|---|---|---|---|---|
| Sneezing | | | | | | | | | | |
| Sore or Scratchy throat | | | | | | | | | | |
| Difficulty swallowing | | | | | | | | | | |
| Teary or watery eyes | | | | | | | | | | |
| Trouble breathing | | | | | | | | | | |
| Chest congestion | | | | | | | | | | |
| Cough | | | | | | | | | | |
| Chest Tightness | | | | | | | | | | |
| Wheezing | | | | | | | | | | |
| Felt nauseous | | | | | | | | | | |
| Vomiting | | | | | | | | | | |
| Diarrhea | | | | | | | | | | |
| Stomach ache | | | | | | | | | | |
| Lack of appetite | | | | | | | | | | |
| Headache | | | | | | | | | | |
| Felt dizzy | | | | | | | | | | |
| Decreased energy/Sleeping more than usual | | | | | | | | | | |
| Clingy/Fussy | | | | | | | | | | |
| Body aches | | | | | | | | | | |
| Fever (temperature 100.4 °F or higher) or Chills or shivering | | | | | | | | | | |
| Altered/lost sense of smell | | | | | | | | | | |
| Altered/lost sense of taste | | | | | | | | | | |
| Did the participant take any medication for their symptoms today? (Enter 0 for No and 1 for Yes) | | | | | | | | | | |

3. What date did the participant return to their usual activity level? (MM/DD/YY)

4. How many days did the participant miss school or daycare?

5. Did the participant seek medical care for their illness through any of the following? If yes, please list the number of visits they had for each of the listed care types:
   a. **Primary care appointment**
      i. Yes
         1. Number of Visits:
      ii. No
   b. **Urgent care appointment**
      i. Yes
         1. Number of Visits:
      ii. No

   c. **Emergency Department**
      i. Yes
         1. Number of Visits:
      ii. No

6. Was the participant admitted to the Hospital?
   a. Yes
      i. Number of Days:
      ii. Did the participant spend any time in the Intensive Care Unit (PICU or ICU)?

            1. Yes
                a. How many days?
            2. No
        iii. Did the participant require oxygen?
            1. Yes
                a. How many days?
            2. No
    b. No, the participant was NOT admitted to the hospital.

7. How many other people live in the home with the participant?
    a. How many people in the household are active duty military?

8. Did anyone living in the home with the participant develop symptoms of a viral infection **AFTER** the study participant?
    a. Yes
        i. How many were active duty military?
        ii. How many were civilians?
        iii. How many under the age of 18?
        iv. How many over 65 years?

    b. No

9. How many total days were one or more care providers out of work as a result of the participant's illness?
    a. For active duty military care providers, how many missed duty days occurred?
    b. For non-military care providers, how many days of work were missed?
