# Supplementary material for: Pediatric respiratory co-infection and immunologic response: peds recon study protocol
Source: Pediatr Res. 2025 Oct 24;99(6):2206–12. doi: 10.1038/s41390-025-04509-9 (PMC13433244; doi:10.1038/s41390-025-04509-9)
Supplement: Supplementary file 2 [file 41390_2025_4509_MOESM2_ESM.pdf]

Supplemental Table S2: Respiratory Pathogens Detected by Research-Grade Multiplex Polymerase Chain Reaction[1] utilized in the Pediatric Respiratory Co-infection and Immunologic Response Study (Ped RECON)

**Pathogen Type**	**Pathogen Name**
Viruses	Adenovirus<br>Coronavirus 229E<br>Coronavirus HKU1<br>Coronavirus NL63<br>Coronavirus OC43<br>Severe Acute Respiratory Syndrome Coronavirus 2 (SARS-CoV-2)<br>Human Metapneumovirus<br>Human Rhinovirus/Enterovirus<br>Influenza A<br>Influenza A/H1<br>Influenza A/H3<br>Influenza A/H1-2009<br>Influenza B<br>Parainfluenza Virus 1<br>Parainfluenza Virus 2<br>Parainfluenza Virus 3<br>Parainfluenza Virus 4<br>Respiratory Syncytial Virus (RSV)
Bacteria	Bordetella parapertussis<br>Bordetella pertussis<br>Chlamydia pneumoniae<br>Mycoplasma pneumoniae

[1] The **BIOFIRE® Respiratory 2.1 (RP2.1) Panel** is designed to detect an array of respiratory pathogens, including 18 viruses and 4 bacteria. The overall sensitivity and specificity of the panel are reported at 97.1% and 99.3%, respectively.

REF**: bioMérieux.** BIOFIRE® Respiratory Panels. Available at: https://www.biomerieux.com/us/en/our-offer/clinical-products/biofire-respiratory-panels.html. Accessed March 12, 2025.
